# In constant search of the good: a qualitative study into insiders’ perspectives on living well with dementia

**DOI:** 10.3389/fpsyt.2024.1285843

**Published:** 2024-02-01

**Authors:** Gili Yaron, Franka Bakker, Simone de Bruin

**Affiliations:** Research Group ‘Living Well With Dementia’, Windesheim University of Applied Sciences, Department of Health and Well-being, Zwolle, Netherlands

**Keywords:** dementia, living well, good life, quality of life, well-being, care ethics, qualitative study

## Abstract

**Introduction:**

The new concept of ‘living well with dementia’ is currently gaining traction in practice, policy, and research. However, people with dementia and their carers’ own understanding of this concept has not received much scholarly attention. This is because empirical studies into living well with dementia are predominantly quantitative; there are only a few qualitative studies on this topic. This study therefore sets out to investigate what living well means for ‘insiders’ in an everyday context.

**Methods:**

To explore insiders’ own perspectives on living well with dementia, we conducted interviews and focus group discussions with 21 dementia stakeholders. The study included ten individuals with mild-to-moderate dementia living at home, five family carers who are or have been involved in the care for a person with dementia, and six health and social care professionals.

**Results:**

Living well with dementia, for our study participants, revolves around shaping their daily lives according to their values. In this sense, living well with dementia is no different than without. As it involves the values of the person with dementia and those of their social network, living well is both an individual and a collective concern. Having dementia undermines people with dementia's ability to substantiate their values, but it is still possible to live well. As they attempt to shape living well with dementia, respondents encounter tensions within the social network and within the person with dementia. To handle these tensions, they work for mutual attunement by using sensitivity and switching between leading and following in social interactions.

**Discussion:**

Living well with dementia in a daily context is a dynamic process in which people with dementia, family carers, and professionals constantly seek the good together. This insight contributes to a better understanding of stakeholders’ ongoing, invisible efforts to mutually attune. It may also help bypass dichotomizing approaches to dementia. Finally, it opens up new venues for research into reciprocity in the care collective. The article closes with recommendations to improve dementia care and support in light of these findings.

## 1 Introduction

Traditionally, biomedical approaches have dominated understandings of dementia, emphasizing negative consequences such as loss of function, decline, and death. This fatalistic perspective typically uses negative dementia frames, fueling the so-called ‘tragedy discourse’ (1–4). The last thirty years have seen the rise of another, more appreciative perspective in research, policy, and practice: one that centers supporting autonomy, dignity, inclusion, meaningfulness, participation, and well-being. On the one hand, this ‘appreciative turn’ includes the ‘personhood movement’, which focuses on enduring personhood, the lived experiences of people with dementia, and social relationships (5–9). On the other hand, appreciative approaches emphasize positive language, adaptability, and remaining strengths and abilities (10–12).

As part of the latter, more positive perspective on dementia, research publications increasingly feature the new concept of ‘living well with dementia’. Conceptual articles addressing living well with dementia advocate the new concept’s merits (including destigmatisation and empowerment) while exploring its practical implications or theoretical underpinnings (4, 9–12). Critics, by contrast, warn against the pitfalls of an overly positive outlook on dementia, which may serve to deny the suffering of affected individuals and make them personally responsible when ‘failing’ to live well (1, 2).

The bulk of empirical studies deploying the concept of living well take a quantitative approach, determining people with dementia’s and their family carers’ ability to live well, or the impact of various interventions on this ability (13–19). To generate and interpret data, these studies use existing measures such as quality of life, well-being, and life satisfaction. The concept is thereby implicitly assumed to be a theoretical rather than everyday notion. This approach therefore yields little insight into how people with dementia and those involved in their care understand living well in the context of everyday life.

Qualitative approaches may help disclose insiders’ perspectives regarding living well with dementia. However, the limited number of qualitative studies on living well with dementia tend to use this concept as a synonym for quality of life or coping (20, 21). We currently know of only four qualitative studies explicitly engaging with respondents’ own ideas regarding living well with dementia, but these focus on professionals in residential care, public perceptions of dementia, and the Korean context (22–25).

The question therefore remains: how do people with dementia and their carers understand living well with dementia in an everyday context? Drawing on a qualitative study with various dementia stakeholders, this paper aims to explore their ‘insider’ perspectives on living well. The study involved people with mild-to-moderate dementia who live at home, family members who are or have been involved in the care for people with dementia, and professionals.

In the end, our goal is to contribute to a more nuanced understanding of what living well with dementia entails, without falling back on overly negative or positive frames. As will become clear, this paper offers three distinct contributions. On an empirical level, it expounds various stakeholders’ views regarding living well with dementia in a daily context. On a conceptual level, it further develops the concept of living well as a dynamic, collective process, and proposes directions for further research into its reciprocal aspects. On a practical level, the paper provides recommendations for improving dementia care and support.

## 2 Methods

### 2.1 Study design, participants, and recruitment

To understand stakeholders’ perspectives on living well with dementia, we conducted a qualitative study between March 2021 and March 2022 in the Netherlands. Data were collected through fourteen interviews and two focus group discussions. Participants were people with various types of mild-to-moderate dementia living at home (n=10), family carers who care or have cared for a person with dementia (n=5), and health and social care professionals working with people with dementia at home as well as in adult day-services centers (n=6) (see **Tables 1**, **2** for participants’ characteristics).

**Table 1:** Overview of research participants (interviews).

Respondent number	Respondent status	Interview type	Age	Gender	Length interview
1	Professional: case manager dementia	Semi structured	28	Female	60 min
2	Person with dementia Partner (dyad interview)	Narrative, semi structured	87 82	Male, female	60 min
3	Family carer (partner, see interview 2)	Semi structured	82	Female	110 min
4	Professional: coordinator adult day-services center	Semi structured	50	Female	60 min
5	Professional: municipal social worker	Semi structured	25	Female	56 min
6	Professional: home care counselor	Semi structured	23	Female	68 min
7	Professional: coordinator adult day-center	Semi structured	56	Female	61 min
8	Family carer (daughter)	Semi structured	53	Female	56 min
9	Family carer (daughter)	Semi structured	45	Female	69 min
10	Family carer (daughter)	Semi structured	50	Female	96 min
11	Professional: owner home care agency	Semi structured	53	Female	58 min
12	Family carer (son, see interview 13)	Semi structured	72	Female	53 min
13	Person with dementia Son (dyad interview)	Narrative, semi structured	90 72	Female, male	30 min
14	Person with dementia Daughter (dyad interview)	Narrative, semi structured	Undisclosed	Female, female	45 min

**Table 2:** Overview of research participants (focus groups).

Respondent number	Respondent status	Age	Gender
15	Person with dementia	72	Male
16	Person with dementia	73	Male
17	Person with dementia	81	Male
18	Person with dementia	74	Female
19	Person with dementia	88	Female
20	Person with dementia	74	Male
21	Person with dementia	74	Male

The choice to include people with mild-to-moderate dementia was motivated by the need for participants to be able to reflect upon the notion of living well with dementia. Participants were recruited using convenience sampling. The authors first contacted professionals in their network, asking them to participate in the study. In addition, we asked these professionals to recruit people with mild-to-moderate dementia living at home and their family carers. Since the study focused on the subjective meanings attributed to living well with dementia in general, we did not collect information on the type of dementia, year of diagnosis, or cognitive function.

### 2.2 Ethics

The Medical Ethical Committee of [Isala hospital, Zwolle, the Netherlands] approved our study proposal (study number [210718]). All participants with dementia were at early stages of dementia and were living in the community independently. In the recruitment stage, prospective participants received an information sheet on the study’s purpose, expectations, data generation and data management, exit-option, and confidentiality. In addition, they received a copy of the informed consent form in advance. These materials made use of dementia-friendly, accessible language (B1 language proficiency level). The research team ensured included participants with dementia were able to fully engage in conversation and comprehend the information sheet and consent procedure. Information on the study and consent was provided again at the start of each interview and focus group, after which participants all signed for informed consent. During interviews and focus groups with people with dementia, the researchers took care to regularly check participants’ well-being, and accommodate their abilities by keeping the conversation short or setting breaks.

### 2.3 Data collection

The interviews lasted 30–110 minutes and were conducted by a student affiliated with our research group. Interviews with people with dementia and family carers took place at their homes, professionals were interviewed over the phone or online. Professionals and family carers were interviewed using a semi-structured approach. To guide the interviews, the interviewer used an topic list designed in advance. To enable participants to articulate their own perspectives on living well with dementia, each interview started inductively by asking about the personal meaning of this notion. Following topics were based on a synthesis of gerontological theories regarding ‘aging well’, since the theoretical underpinnings for ‘living well with dementia’ are still limited. Gerontological theories approach aging as lifelong process involving interactions between individuals and their social and physical environment. Various values (e.g. autonomy, pleasure, good relations) and resources (e.g. health status, various literacies, financial security) are key for aging well. To draw out these aspects in the interviews, topics addressed the role of people with dementia themselves, their social environment (e.g. family carers, neighbors, friends, professionals), their physical environment (including health and social care provisions), values, and resources in living well with dementia.

Interviews with people with dementia were divided into a semi-structured and a narrative part. For the semi-structured part, the interviewer used the topic list described above. For the narrative part, the interviewer used visual aids to help interviewees articulate their views, despite their cognitive impairments. Participants were offered a stack of random images (e.g. landscapes, everyday objects, people), and asked to select three that represent living well to them and elaborate on these. Interviews with people with dementia included a family carer to ensure respondents felt safe.

The focus group discussions were held at a later stage, and included seven people with dementia. These group conversations were organized to guarantee the perspectives of a sufficient number of people with dementia were represented in the study, allow for new insights arising from reflection among peers, and collect people with dementia’s feedback on an early version of the data analysis. These focus groups lasted 98 and 72 minutes respectively and took place at an adult day-services center for people with dementia. The first author led the discussions together with a chaplain with expertise in existential issues in dementia, using an adapted version of the interview topic list. Data collection reached saturation during the second focus group. The first author visited the day center thrice before the focus groups commenced to get a sense of the setting and ensure respondents’ comfort. All conversations were audiotaped and transcribed verbatim; GY took field notes throughout the data collection and analysis.

### 2.4 Data analysis

During the analysis, we gradually discovered the role of values, tensions, and mutual attunement in living well with dementia. To understand these findings we took direction from the ethics of care, a scholarly tradition that has long analyzed care relations (26–28). Leading care ethicist Joan Tronto defines care broadly as “*a species activity that includes everything that we do to maintain, continue, and repair our “world” so that we can live in it as well as possible*” (26). The ethics of care views health and social care, in particular, as an ongoing and ever-changing relational process. This process involves mutual attunement and attentiveness between care providers and care receivers—who are often vulnerable and dependent (29–31). Using care ethics as an analytical lens allowed us to forefront participants’ values, unpack various inherent tensions, and understand how they negotiate such tensions in daily care.

Analyzing the data, we followed the steps of thematic analysis (32). Taking an iterative approach, the first author and two colleagues read and open coded five interview transcripts. GY then devised a coding tree and used it to deductively assign codes to relevant passages in all interviews, adding additional inductive codes when identifying new topics. Analyzing the focus group transcripts and field notes, she checked and enriched the previously identified codes. Next, she further refined and reduced all the codes generated by grouping them together into categories, thereby yielding candidate themes. Going back and forth between the codes, candidate themes, and written article, all authors then further refined the analysis together. During this process, we discussed the scope, coherence, and distinctness of the themes until reaching consensus. All authors are experienced qualitative researchers. GY contributed insights from care ethics while FB and SdB contributed knowledge on dementia and insights from the dementia research literature.

## 3 Results

Analyzing the interviews and focus groups, we generated three overarching themes regarding the meaning of living well with dementia in the context of daily life. Firstly, ‘living well’ encompasses several values that receive concrete substance in participants’ daily practices. Secondly, as they attempt to shape living well with dementia, respondents encounter two tensions. Thirdly, negotiating these tensions asks that people with dementia and their carers work to mutually attune.

### 3.1 Giving substance to various ‘goods’ in everyday life

Living well with dementia, for participants in all three groups (people with dementia, family carers and professionals), entails shaping daily activities according to the values of all involved. Although people with dementia’s condition undermines their ability to substantiate their values, they can still live well. This is linked to the ability to create happy moments and appropriately relate to difficulties and limitations.

#### 3.1.1 ‘Goods’ as values in practice

Asked what ‘living well with dementia’ means to them, participants with dementia provided concrete examples of what they consider ‘the good’ in everyday life. These include: walking in the dunes, enjoying food, spending time with friends and family, sleeping sufficiently, playing music, living in a pleasant, clean and safe house, helping others, and travelling independently. As one respondent said:

“(…) That I can still ride my bicycle and drive a car. I can go where I wish (…) If I want to [I can] visit my sister-in-law.” (person with dementia, interview 13)

Importantly, respondents in all three groups emphasized that living well with dementia is not very different than for anyone else; it is “nothing spectacular [and involves] simple things”, one person with dementia said (interview 2). Moreover: there is no ‘blueprint’ for living well:

“There is almost no formula to be found regarding what is good. Just the regular human things that usually take place as a matter of course, and that do no longer go so smoothly for people with dementia.” (professional, interview 4)

Living well, it appears, is a subjective enterprise that receives various personal meanings.

All the examples participants enumerated are in fact all expressive of their values; the abstract ‘good’, therefore, seems to contain various more specific ‘goods’. People with dementia emphasized independence, pleasure, good health, companionship, meaningfulness, autonomy, and accessibility of services. Moreover: some indicated it was important that their carers were not overburdened.

Carers’ own values were also a recurring topic in the interviews. Carers’ discussed three types of values: a) things they considered important for themselves (self-care, good relationships, professionality), b) things they considered important in the context of dementia care (hygiene, safety, genuine attention, respect, familiarity), and c) things they considered important for the person with dementia (health, pleasure, autonomy). An example of the second type of care-related values can be found in the story of one family carer. Speaking of how much her father—a former physician—appreciated medical professionals’ respectful manner during his wife’s deathbed, she explicitly linked this to the values of ‘being taken seriously’, ‘security’, and ‘familiarity’:

“It was very good for him, that he was taken very seriously and received extensive explanations why [a certain treatment] wasn’t possible anymore and such. It was his own profession. (…) I think that is very dear to him, that [he] finds a sense of security, familiarity in it.” (family carer, interview 10, emphasis added)

Since both people with dementia and carers expressed concerns about the ‘goods’ of significant others, values appear to be somewhat intertwined. And indeed, several participants in all three groups stressed that living well pertains both to the person with dementia and their social network. This network may be comprised of partners, family members, friends, and neighbors, but also professionals. In that sense, living well with dementia seems to be a collective as well as individual concern—and involves attending to the ‘goods’ of all.

#### 3.1.2 The possibility of living well: creating the good and relating to the bad

Various respondents in all three groups agreed that people with dementia’s ability to live as they see fit may be undermined by their condition. This is due to problems with daily functioning. In addition, dementia’s progressive nature, erratic development, and bleak outcomes may bring about difficult emotions (e.g. grief, shame, worry). Such emotions sometimes arise due to others’ responses:

“[My] brothers and sisters (…), I deliberately told them everything that I wanted to get off of my chest. (…) Also about that dementia. And there is nobody who ever asks about it. And I do find this to be very strange, I do. They think: ‘dementia? (…) [people with dementia] don’t really count anymore’. (…) Or they just listen to you and they don’t say anything at all in response. (…) [So] I just don’t tell much anymore.” (person with dementia 19, focus group 2)

Here, the stigma surrounding dementia rather than dementia itself is detrimental.

Nevertheless, most respondents who raised this issue asserted that living well with dementia is still possible. However, their stories imply this is conditional on the type of dementia and its development, as well as on external circumstances and stakeholders’ personal attitudes. External circumstances consist in the availability of personal resources (e.g. a social network, health status, money), accessible care and support, clear information on dementia, provisions in the neighborhood (e.g. adult day-services centers, transportation), and a dementia-friendly society.

Personal attitudes consist in stakeholders' disposition (e.g. being friendly, accepting help). Participants’ stories suggest that two attitudes are especially important for living well: a) the ability to actively create happy moments, and b) the ability to appropriately relate to difficulties and boundaries. Actively creating happiness involves making and cherishing meaningful moments:

“What I do find to be important (…) is to dwell on those moments (…) like a Mother’s Day, birthday, and Christmas. Experience them much more intensely and also record [them] with pictures or rituals.” (family carer, interview 9)

Similarly, other family carers and professionals discussed how they try to avoid confronting people with dementia with their impairments, while ensuring they experience moments of pleasure, competence, and success (e.g. drinking a glass of wine after dinner, managing a challenging game, showcasing their photographs). These participants maintained that a sense of accomplishment, in particular, help foster people with dementia’s self-worth.

But living well also entails appropriately relating to difficulties and limitations. Participants in all three groups valued recognizing challenging moments and emotions without playing them down. Thus, many underscored the importance of accepting one’s impairments, growing dependency, and inevitable decline. In addition, respondents indicated dementia may bring two clear boundaries that should be acknowledged: the moment when the person with dementia must move to a nursing home, and death. As one professional said: “A good life is perhaps also a good ending” (interview 7).

### 3.2 Tensions within

Participants’ daily efforts to give concrete shape to the values they associate with living with dementia take place within a complex social network. In the process, they reported encountering two tensions: a) within the social network, and b) within the person with dementia.

#### 3.2.1 Tensions within the social network: conflicting values, truths and needs

Stakeholders within the person with dementia’s extended social network—including that person—may experience conflicts over opposing values, truths, and needs. The first conflict has to do with stakeholders preferring different values. One interviewee shared that her mother with dementia’s social housing agency claimed the value of privacy when explaining why they did not contact the daughter after the mother had repeatedly flushed her underwear through the toilet. The daughter, however, felt her own value of engagement was not considered (interview 9). Other respondents described conflicts between cleanliness (ensuring a parent with dementia showers regularly) and autonomy (the parent’s wish not to shower) (interview 10). Significantly, the family carer in this case also experienced an internal value conflict: she found it important to ensure cleanliness *and* respect her father’s autonomy. A third example concerns the tension between tolerance (making allowances for a woman with dementia’s challenging behavior during choir rehearsals) and doing no harm (the choir members’ wish to rehearse peacefully) (interview 9). A related issue has to do with professionals’ conduct. As the stories of professionals imply, professional standards in fact embody values such as ‘efficiency’, ‘safety’, and ‘accountability’. However, these may collide with other ‘goods’. For instance, efficiency requires that a home care professional keeps a strict schedule, which may not fit with a family carer’s value of flexibility (interview 8).

The second conflict is associated with stakeholders’ varying perspectives on reality. Thus, not everyone in the social network may believe that the person with dementia has a medical condition:

“(…) my older brother and sister (…) talk about [our mother’s] memory issues as being dementia. I have volunteered with older people with dementia (…) that’s something completely different. That designation, we don’t agree on it.” (family carer, interview 14)

Her mother added:

“I sometimes forget something, but that doesn’t mean you’re becoming demented [sic]. That’s something else entirely.” (person with dementia, interview 14)

Likewise, the person with dementia and significant others may have a different perception of events. For instance, during one dyad interview a person with dementia insisted he avidly follows current political developments, but his wife pointedly assured the interviewer this is no longer the case (interview 2). Several professionals stressed such clashing ‘truths’ may give rise to friction (e.g. over the person with dementia’s ability to drive or live independently).

The third conflict is linked to opposing needs of individuals within the network, in particular those of people with dementia and their family carers. As one family carer shared:

“(…) playing Scrabble is very important to [my husband] (…) [so] I would be playing scrabble three times a day sometime, but (…) I have to make sure dinner is served, and the groceries are done. (…) [And] I have to ‘run’ [function] too.” (family carer, interview 3)

Playing Scrabble serves to alleviates the husband’s agitation, but his need clashes with the wife’s need to handle household matters and secure ‘me-time’. Other family carers, too, discussed struggling to combine the care and support for people with dementia with childcare, paid work, and leisure activities. This tension, again, resides both between and within stakeholders. Similarly, various professionals indicated that adult day-services centers can be a mixed blessing: the absence of the person with dementia can provide the family carer with a much-needed break, but some people with dementia cannot abide the hustle and bustle at the center.

Being a communal undertaking, living well with dementia inevitably brings along conflicts associated with real differences within the social network. The tensions between opposing values, truths, and needs results in a daily dilemma regarding the question: should I prioritize my own position, or that of the other person?

Conflicts, of course, are not unique to dementia: they occur in any situation with multiple stakeholders. However, dementia makes such conflicts more pressing because of the inherent vulnerability and dependency it brings:

“(…) you see marriages break down (…). It’s quite a change, they [are] no longer partners. He is the care receiver and I am the care provider (…). [T]here is a certain balance before (…) and you were equal (…) and at a certain point that just diminishes.” (professional, interview 6)

This unequal position means that family carers hold a particularly complex position in disputes, being at once party and arbiter. This point also holds for professionals vis-à-vis family carers who are overwhelmed by caring responsibilities or the often-confusing world of formal provisions.

#### 3.2.2 Tensions within the person with dementia: conflicting selves

The second tension involves discrepancies between the person with dementia’s past and present identity. Speaking of the meaning of living well with dementia, many participants in all three groups stressed the importance of preserving “how someone lived before the diagnosis” (professional, interview 1). Participants mentioned four ways in which they strive to preserve the person with dementia’s past-self. First, such preservation is about maintaining familiar habits and preferences; respondents mention keeping up with cooking and eating favorite dishes together, a life-long routine of playing the piano, and gardening. Family carers and professionals, in particular, all spoke of encouraging such activities, which they see as connected to the person with dementia’s typical preferences:

“[W]e rented a wheelchair. And if the weather is nice this weekend, I want to walk with [my mother] to the petting zoo. Well, she can’t undertake things herself, so I’ll be looking for, what is good for her and what would she like. I know she loves animals and (…) when she could still walk, she used to enjoy going to watch the goats. So I just assume that’s still the case.” (family carer, interview 8)

Second, carers reported that they find it important to keep stimulating the person with dementia to remain active so as to counteract apathy, which they saw as a symptom of dementia. Third, carers keep including the person with dementia in everyday decision-making. Finally, they work to prevent what they see as loss of decorum (e.g. ‘inappropriate’ dress or behavior). These efforts to preserve the past-self speak to carers’ wish to maintain the person with dementia’s unique identity and status as a capable and dignified human being.

At the same time, all respondents also indicated that dementia changes how the affected person deals with others and the world. Alterations may consist of new challenges (e.g. problems with reading or calculating, losing one’s way, difficulties with household tasks). In addition, carers pointed out behavioral changes:

“Someone can change completely in terms of their behavior, develop other interests, become withdrawn. People can become very inaccessible.” (professional, interview 5)

In addition, personal changes may also result from the confrontation with the diagnosis and prognosis. As discussed above, people with dementia indicated they experience difficult emotions in relation to their condition, which affects their overall disposition. By contrast, people with dementia and carers also describe the emergence of positive attitudes such as acceptance, living in the moment, and an appreciation for the little things in life.

People with dementia do not change overnight, but their identity increasingly manifests as a mixture of past-self and present-self: “[These] people are a composition of who they were and who they are now (professional, interview 11). Respondents’ stories imply that the tension between the two ‘selves’ poses two dilemmas linked the question: who is the ‘real’ person with dementia? First, it can be difficult to decide which expressions of the ‘self’ are authentic:

“(…) [Y]ou have to (…) address someone as a person, and the disease, you approach that as additional. (…) [But] you see, [dementia] plays a very clear role (…) because they forget things or exhibit changed behavior, so you can’t fully see it apart.” (professional, interview 7)

This mixture of old and new elements complicates efforts to comply with the preferences of people with dementia. Second, the combination of old and new elements may lead others to expect either too much or too little of the person with dementia. Expecting too much is associated with holding on to the fully capable past self, which may evoke frustration or fear in the person with dementia. Expecting too little is associated with dismissing the person altogether, and no longer trying to understand or engage them. This, many participants indicate, is demeaning and may hasten the dementia’s progression.

### 3.3 Working for mutual attunement

Living well with dementia, as illustrated above, involves various tensions. Participants related they negotiate these in practice, by aligning care activities (e.g. dressing, transportation, social activities), people (e.g. family members, professionals), things (e.g. diagnoses, wheelchairs), and times (e.g. present issues, future scenarios). But negotiating tensions goes beyond practical concerns: it requires that respondents attune to one another by using sensitivity and switching between leading and following.

#### 3.3.1 Developing ‘sensitive feelers’

Attunement entails developing a sensitivity towards the other—whether that other be a person with dementia, family carer, or professional. As one professional said:

“I know someone who goes to this (…) adult day-services center, he freaks out. That man just wants to be alone (…) [We should] look at who someone is and adjust to that. (…) As if [people with dementia] are able to say: ‘I don’t feel like it, so leave me alone. Do we really have to go to the adult day-services center again?’ [Meanwhile], she always does return with a lot of anger. Well, duh. (…) [So] look, right, look at what makes people light up. That twinkle in the eye (…).” (professional, interview 11)

As this quote illustrates, people with dementia can experience difficulties in expressing their needs. Being sensitive to their mood can help others spot these. Similarly, various family carers spoke of a need to have their struggles acknowledged by their general practitioner. Professionals, too, indicated the importance of recognizing when family carers meet the end of their rope. Professionals also discussed being sensitive to colleagues’ need for support, for instance when a young co-worker struggles with sexually uninhibited behavior by a person with dementia. People with dementia, too, indicated they sensitively attune to others’ needs, for example by noticing the burden their family carers experience (focus group 1). Again, it seems that living well is a quality of the collective, pertaining to all stakeholders in dementia care.

Sensitivity also extends to monitoring the effect of activities and interactions. Most carers spoke of using their ‘feelers’ to determine what works and what does not work for those they support and care for. They watch for signs of under- or overstimulation when undertaking familiar activities or introducing new ones. Professionals also carefully feel out whether a person with dementia and their family carers prefer the term ‘dementia’, a more neutral phrase such as ‘memory problems’, or a description such as “your head is not working like it used to” (field notes).

#### 3.3.2 Switching between leading and following

In addition to mutual sensitivity, attunement also involves the dynamic ability to switch between leading the other and following them^1^. Leading consists of subtly taking the other along with daily activities, plans, and ideas. Professionals and family carers, for instance, tell the person with dementia how a certain care procedure will take place, clarify why they think an alarm is required, and reassure that forgetting things does not mean one does not count anymore. Carers also explain to others (e.g. fellow singers in a choir) why the person with dementia acts as they do. One family carer elaborated on how she uses humor to handle her father’s resistance to showering:

“My father dislikes taking showers. (…) but there are limits: he obviously does need to stay clean. (…) [A]nd the home carer would come and I’d say: ‘dad, you can take a nice shower again’. Then he’d look at me and say: ‘no way, you go’. We used to make that into something of a running gag. (…) But then I’d say: ‘dad, you don’t want to become a stinky old man, do you’, that way he would [take that shower].” (family carer, interview 10)

Other examples of leading are: tempting the person with dementia to go to the adult day-services center by presenting it as ‘work’, activating the person with dementia by offering a choice between dishes, and stimulating interaction by binging up a recent visit by relatives. Several professionals also mentioned taking the initiative to mediate between the person with dementia and family carers when tensions arise. Significantly, people with dementia take the lead when they minimize the challenges they face or refrain from asking for help from carers, due to concerns about burdening their carers (focus group 1).

Following, by contrast, revolves around facilitating others’ (implicit) values, truths, needs, or identities. Thus, people with dementia follow by allowing specific types of care and support their carers find important, or going to an adult day-services center when their family carer is exhausted. Professionals and family carers choose to not make a fuss over every single mistake by the person with dementia, or go along with their version of the truth: “There are a number of basic steps, like never going against [the person with dementia], [and accepting] that the person with dementia is always right, even if it makes no sense (professional, interview 5). Professionals and family carers also spoke of catering to specific talents of people with dementia:

“I’m now [working with] a very technical man, I’m trying to get him to a [adult day-services center] where he can get technical activities. And yes, then he will be stimulated in his talent (…) and in his expertise.” (professional, interview 6)

Similarly, another professional working at an adult day-services center asked a visitor with dementia to showcase his photographs at the location, adding that she will “only accept really good ones” (field notes). In this way, she provided him with a tailored project and an opportunity to connect to the location, while supporting his self-worth.

Following may also entail avoiding unnecessary or harmful conflicts. Thus, one family carer described how she gets her father with dementia to change his dirty underwear and socks without constantly reminding him about this—reminders which, she senses, make him uncertain. When the father is in the shower, the daughter surreptitiously replaces his cast-off underwear with clean ones. This way, she manages to give practical shape to both their perspectives on ‘living well’: her own value of ‘cleanliness’, as well as his value of ‘dignity’ (interview 10). Respondents mention similar examples such as hanging curtains in front of doors or letting the hedge grow in front of the garden entrance to ensure people with dementia do not go off wandering while preventing unnecessary conflicts^2^. Participants who are carers also discussed giving others time and space to handle challenging situations in their own pace. One professional shared how she actively “leans back” if the person with dementia is not ready to receive support:

“[This woman] is totally losing her overview of things now (…). And that causes so much agitation (…). And you try to put [support] in motion but (…) [her] autonomy, that’s very hard to let go. (…) Yes, you need to do things in her pace. In this, I see from a ten-kilometer distance that she can do a lot but also a lot not. So yeah, that’s very difficult, but you let someone muck about for a while” (professional, interview 1)

Indeed, following implies leading and leading implies following—since both involve the relationship between dementia stakeholders.

## 4 Discussion

### 4.1 Constantly seeking the good together

This paper set out to explore how people with dementia, their family carers, and professionals understand living well with dementia in the context of everyday life. As our analysis demonstrated, ‘living well’ for people with dementia is not very different than for others. A subjective enterprise in which all stakeholders give substance to their values, living well entails intricate interplays between people with dementia, family carers, and professionals. Thus, living well does not only pertain to the person with dementia, but also to the entire ‘care collective’. Having dementia does undermine people’s ability to substantiate their values, but it is still possible to live well. This is linked to the ability to actively create happy moments and appropriately relate to difficulties and limitations. As stakeholders attempt to shape living well, however, they encounter two tensions, involving a) different values, truths, and needs within the social network, and b) the mix of past and present elements within the identity of the person with dementia. Handling these tensions requires that stakeholders attune to one another by using sensitivity and switching between leading and following. This implies subtle forms of mutual care between people with dementia and their carers. Living well with dementia, we conclude, appears to be a dynamic process, in which people with dementia, family carers, and professionals constantly seek the good together in a complex and changeable situation.

In line with various quantitative studies into living well with dementia, positivity and empowerment seem to be key aspects of living well (13–17). As our analysis showed, actively creating happy moments of pleasure, competence and success is vital in this (theme 1. However, quantitative studies do not unpack the often-subtle ways in which stakeholders accomplish positivity and empowerment. Meanwhile, qualitative studies into living well with dementia rarely address these aspects (33). One notable exception is Driessen’s work on the enactment of pleasure, daily wants, and preferences in dementia nursing homes (34–36).

Moreover: positivity and empowerment are only part of the story. Living well is also about appropriately relating to ‘the bad’: the difficulties and boundaries associated with dementia. Although relating to difficulties is not the primary focus of the living well literature, the management of challenging emotions is part of the concept of well-being and can also be seen as ‘coping’—which some studies on living well address (13, 16, 17, 37). Still, most studies into living well pass over two important aspects herein: the various tensions inherent to everyday life with dementia, as well as the subtle ways in which stakeholders negotiate these. Living well with dementia, we argue, encompasses the full spectrum of human experience. Moreover: it includes everyday negotiation of the tensions that arise as stakeholders seek the good together.

As a dynamic process, living well has a clear temporal dimension; the concept can be understood as a verb, describing a changeable, unpredictable, and ongoing endeavor. Most of the literature on living well takes a static approach in measuring delineated ‘cut-outs’ of selected populations’ well-being, quality of life, and life satisfaction. But living well, as we see it, should be distinguished from ‘the good life’ in so far as the latter implies an atemporal, fixed condition. As Schermer (2003) writes, it remains unclear whether theories on the good life focus on a particular moment, a certain period, or an entire lifespan (38). This unclarity, Schermer concludes, makes is hard to account for “the factor ‘time’” (38). A narrative approach to living well, she suggests, might allow for development and change. Our dynamic understanding of living well proposes exactly such an approach.

Attending to the dynamic character of living well with dementia has three important advantages. Firstly, this foregrounds carers’ constant, communal efforts as they search for the good. Since what ‘living well’ entails is not evident, agreed upon, or easily realized, their efforts are a subtle form of invisible labor: rarely acknowledged as such, but significant and exhausting nonetheless. Many studies into dementia care discuss carers’ experienced burden (39–42) and the importance of supporting their quality of life in this context (43). Vos et al., in particular, indicate family carers require proper recognition of their labor and the challenges they face (40). A significant part of carers’ burden, we suggest, is not caused by what is commonly seen as ‘care work’ (e.g. meeting functional needs or providing emotional support), but by the management of tensions between social actors. Indeed, opposing values and needs may lead to contention in care dyads as well as triads (44–46). Attuning to multiple others in daily life, as we show, involves the ongoing balancing of conflicting values, truths, needs, and selves. A collection of countless invisible little decisions in daily life, this balancing can be a strenuous undertaking.

Secondly, focusing on living well as a dynamic process can help avoid the pitfalls of the ‘tragedy discourse’ while staying clear of the opposite, overly positive and responsibilizing account (1, 2). Living well in an everyday context, as our analysis demonstrated, includes the good as well as the bad. More importantly: it consists in ongoing negotiations between social actors which cannot be easily understood in such dichotomous terms. Living well can thus be framed as a collective effort rather than an individual responsibility, and as a process rather than an outcome one can succeed in—or fail to—achieve.

Thirdly, unpacking the subtle dynamic of leading and following may provide a starting point for further research into reciprocity in dementia care relations. As our study suggests, caring for the good is a two-way street. Importantly, people with dementia allow others to care for them and subtly care for these others. As such, they actively contribute to ‘living well with dementia’, acting as partners in the care collective rather than mere receivers of others’ caring efforts. Further research could explore such reciprocity in care. Asking the question: ‘how do people with dementia care for others?’ could be one way of articulating ‘interesting subject positions’ for people with dementia, as Driessen (2023) puts it —subject positions that allow them to become interesting by opening up unexpected vistas (47).

### 4.2 Strengths and limitations

To our knowledge, this is one of the few qualitative studies into living well with dementia. By highlighting how insiders understand this concept, the study offers a novel perspective on living well with dementia as a collective, dynamic process. One limitation consists in the relatively small number of participants, which is typical to qualitative research. Another limitation concerns the inclusion of people with mild-to-moderate dementia living at home. This choice was motivated by the reflexive setup of the study. Better understanding the meaning of living well with dementia also requires exploring the experiences of people with progressive dementia and those living in residential care. Further research into this concept could include this group.

### 4.3 Recommendations

A dynamic approach to living well may help improve dementia care and support. To better align with stakeholders’ own understanding of living well, the field of dementia care should address, value, and facilitate the daily work of the care collective as it seeks the good together. The training of current and future care professionals, however, mostly centers medical and functional care (48). As a result, professionals experience challenges in managing the rising complexity of the field and adopting new care models that emphasize autonomy, dignity, and well-being (idem). Moreover, care organizations do little to facilitate current professionals in acquiring the necessary training and putting these care models into practice (48, 49).

Rather than focusing on theoretical constructs and measurements, such training requires learning how to recognize and address the tensions between social actors as they seek the good together. Concretely, they must learn to—explicitly and implicitly—ask colleagues, patients, and family carers: what does living well mean to you and which values does this imply? Which tensions do you encounter in the process? And: how can we best handle these? Indeed, as Tuijt et al. (2021) argue, understanding the unique relationship dynamics between people with dementia, family carers, and professionals is vital to the functioning of dementia care triads (50). Questions regarding the good in practice should therefore not be delegated to the domain of religion, spirituality, and chaplaincy, but become embedded within the care process. We therefore recommend that professionals develop and practice competences such as reflexivity, sensitivity, and creativity. To facilitate them in doing so, health and social care organizations can take steps to recognize and value practices of attunement in daily care, and provide time and space for improving on them.

## 5 Conclusion

This paper added to the emerging literature on living well with dementia by exploring how people with dementia, family carers, and professionals understand living well with dementia in an everyday context. Living well with dementia, we conclude, is a dynamic process in which stakeholders constantly seek the good together. This suggests living well pertains both to the individual with dementia and to the ‘care collective’ as a whole. Attending to the dynamic and collective dimensions of living well contributes to a better understanding of the often-invisible efforts of dementia stakeholders as they negotiate tensions in daily life. This insight can also help bypass dichotomizing approaches to dementia, and opens up new venues for research into the reciprocal aspects of care in dementia. Finally, the paper offers a starting point to improve professional dementia care and support by addressing questions regarding ‘the good’ in practice.

## Data availability statement

The datasets presented in this article are not readily available because all the data generated, used and analyzed during the current study are stored on the Windesheim secured server, and are accessible to the authors only. These datasets cannot be completely anonimized; in order to protect research participants’ privacy, these data are therefore not publicly available. The datasets are available from the corresponding author on reasonable request, but requests for viewing and/or using the data will not be automatically granted. Rather, such requests will be handled on a case-by-case basis. Requests to access the datasets should be directed to g.yaron@windesheim.nl.

## Ethics statement

The study involves human and was approved by the Medical Ethical Committee of Isala hospital, Zwolle, the Netherlands (file number 210718). The study was conducted in accordance with the local legislation and institutional requirements. Written informed consent for participation in this study was provided by the participants or their legal guardians/next of kin.

## Author contributions

GY: Data curation, Formal Analysis, Investigation, Methodology, Project administration, Validation, Writing – original draft, Writing – review & editing. FB: Conceptualization, Investigation, Methodology, Project administration, Supervision, Validation, Writing – review & editing. Sd: Conceptualization, Investigation, Methodology, Project administration, Supervision, Validation, Writing – review & editing.

## Footnotes

1 Professionals often speak of ‘moving with’ the person with dementia or family carers in this context.

2 This type of practices is widespread but contested, since they involve a degree of deception. Indeed, leading and following may easily derail, some professionals and family carers indicate. Leading may turn into paternalism, manipulation, or coercion. Following may become neglect or dishonesty.
